# Comparison of Continuous Subcutaneous Insulin Infusion and Multiple Daily Injections in Pediatric Type 1 Diabetes: A Meta‐Analysis and Prospective Cohort Study

**DOI:** 10.3389/fendo.2021.608232

**Published:** 2021-03-02

**Authors:** Xu Wang, Xue Zhao, Danrong Chen, Mingzhi Zhang, Wei Gu

**Affiliations:** ^1^Department of Endocrinology, Children’s Hospital of Nanjing Medical University, Nanjing, China; ^2^State Key Laboratory of Reproductive Medicine, School of Public Health, Nanjing Medical University, Nanjing, China; ^3^Key Laboratory of Modern Toxicology of Ministry of Education, School of Public Health, Nanjing Medical University, Nanjing, China

**Keywords:** type 1 diabetes, children, multiple daily insulin injections therapy, continuous subcutaneous insulin infusion, China

## Abstract

**Background:**

The incidence of pediatric type 1 diabetes (T1D) is increasing worldwide, and the appropriate choice of therapy regimens is important for children, especially in developing countries with inadequate resources.

**Methods:**

We conducted a design combining meta-analysis and prospective cohort study. In meta-analysis, 14 studies involving 69,085 TID cases reported glycosylated hemoglobin (HbA_1c_) levels, including 48,363 multiple daily insulin injections therapy (MIT) and 20,722 continuous subcutaneous insulin infusion (CSII). In our prospective cohort study, TID cases were recruited from a tertiary children’s hospital, and randomly divided into Group MIT and Group CSII. After the 4-year follow-up, the effects of MDI (n = 112) and CSII (n = 76) therapy on glycemic control, long-term complications, as well as the growth and pubertal development were explored.

**Results:**

Compared to CSII in TID, HbA_1c_ levels in MDI (WMD = 0.21, 95% CI: 0.20 to 0.23) were increased significantly in meta-analysis. Among 188 clinical cases, mean age at recruitment was 7.55 (SD 2.91) years. Duration of TID was 4.23 (SD 2.61) years. 50.53% (n = 95) of them were boys. The 4-year follow-up showed that children’s HbA_1c_ was 0.67 (95% CI −1.28, −0.05) % lower in children with CSII compared to children with MDI in multivariable regression models with adjustment for potential confounders (children’s age at follow-up, duration of TID, gender, birthweight, parity, and delivery method). CSII was associated with 2.31 kg higher in children’s weight (95% CI 0.59, 4.04) in the adjusted model. No difference was found in peripheral nerve and fundus consequences as well as the status of obesity and thin and pubertal development between CSII and MIT.

**Conclusion:**

CSII might be associated with better glycemic control and better effect for children growth development. No higher risks of long-term complications and delayed pubertal development were observed in CSII. Our findings provided evidence for a better therapy regimen for T1D in children, nevertheless, they need to be validated by a larger sample size study.

## Introduction

Epidemiological and experimental studies have shown that type 1 diabetes (T1D) and its complications can affect human health. Children and adolescents are at high risk of TID. During 2010–2013, the estimated incidence of TID was 1.93 under the age of 14 in China per 100,000 person years, and this number is expected to increase continually (1, 2). TID is caused by absolutely inadequate insulin production due to the damage to the beta cells of pancreas, and requires lifelong insulin treatment. Several insulin types (rapid, short, intermediate, and long-acting) and infusion methods [multiple daily insulin injections therapy (MIT) and continuous subcutaneous insulin infusion (CSII)] can be used to treat TID.

The use of insulin pumps for CSII therapy among patients with TID increased from 0.6% to 1.3% in 1995 to 44% to 47% between 2012 and 2016 (3). In 2018, American Diabetes Association (ADA) Guidelines recommend the usage of CSII in adolescents with TID (4). However, in the field of pediatrics, the conclusion of comparison of the effects of MIT and CSII for TID were not consistent. Some studies showed that CSII therapy has more advantages in glycemic control among children (3, 5, 6), while other studies showed that CSII therapy is not more effective than MIT (7–9).

Furthermore, regarding MIT and CSII, cares should be taken as to the possible risk of complications. More importantly, for children, attentions should be paid to the impacts of different therapy regimens on growth and development. However, there is little research on this topic in China. Therefore, on the basis of literature review, first, a meta-analysis was conducted to compare MIT and CSII in achieving glycosylated hemoglobin (HbA_1c_) control in pediatric TID. Second, a prospective cohort study was designed to explore the effects of MIT and CSII on glycemic control, acute and chronic complications, as well as the growth and pubertal development in children.

## Methods

### Meta Search Strategy

We searched the relevant literature in four electronic bibliographic databases: PubMed, Cochrane Library, Embase and Web of Science. The published literature was searched until June, 2020. The following search terms were used in the databases:

#1 (children) OR (child) OR (childhood) or (pediatric)

#2 (Type 1 diabetes) OR (T1D)

#3 (insulin injection) OR (insulin infusion) OR (MIT)

#4 (insulin pump) OR (continuous subcutaneous insulin infusion) OR (CSII)

#5 (glycated hemoglobin) OR (GHb) OR (glycosylated hemoglobin) OR (HbA_1c_)

#6: #1 AND #2 AND #3 AND #4 AND #5

We selected studies which:

1. The full text of literature is available;
2. The subjects were children, not experimental animals;
3. Explicitly specified the substance that children were diagnosed as TID;
4. The data showing HbA_1c_ concentration in both MIT and CSII therapy were provided;
5. There were no defects in research design and high literature quality.

**Figure 1:**
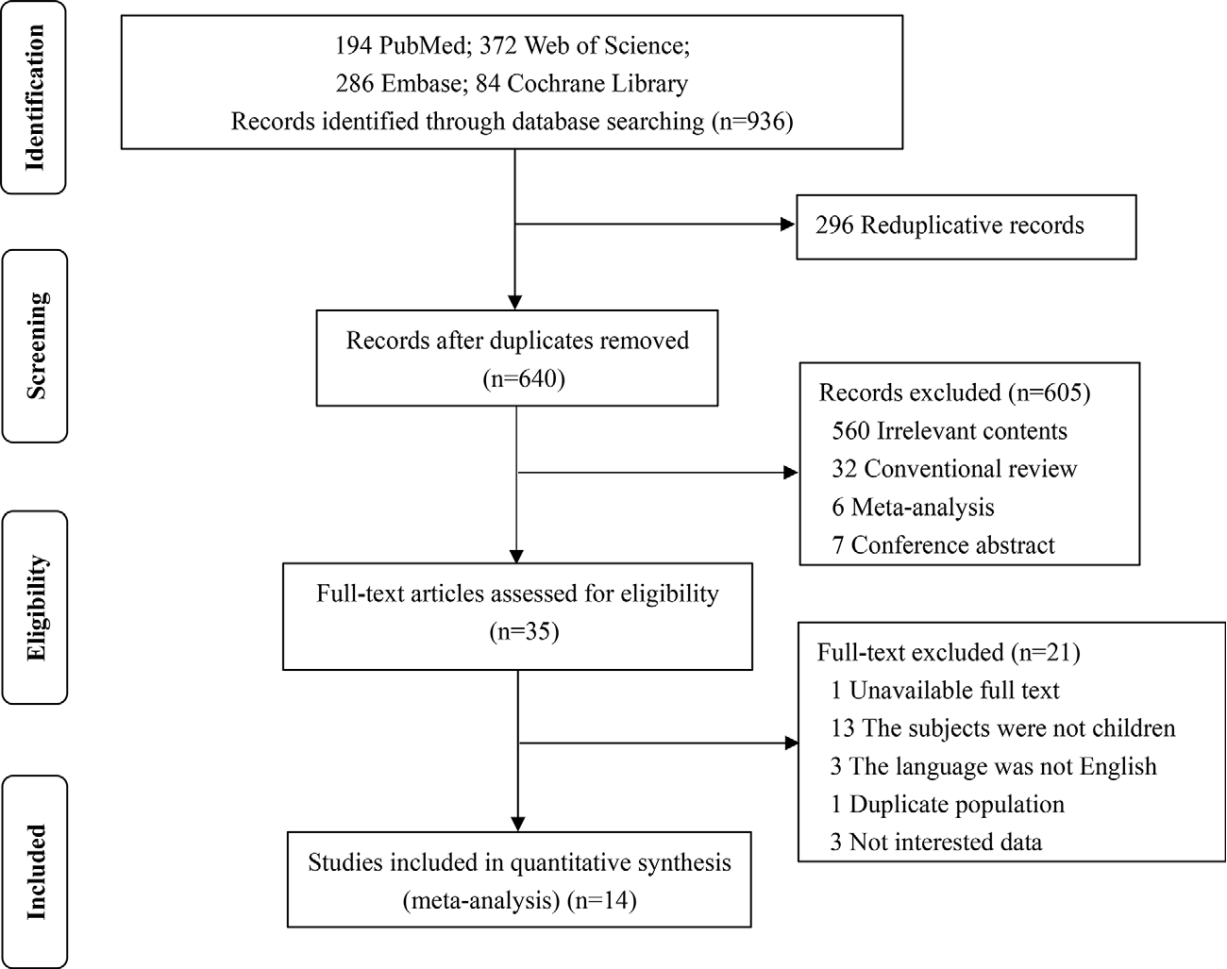
Flow diagram of literature screening.

Two assessors screened and identified the literature. Any disagreement of them was settled by discussion with a third evaluator (**Figure 1**). According to the Newcastle Ottawa Scale (NOS) standard (10), the quality of each literature was evaluated and total number of stars greater than or equal to six as appropriate literature (NOS ranges from 0 to 9 stars) were selected. The information of each on the 14 literature was extracted (3, 5–9, 11–18), including author name, published year, research time, region, sample size, age, gender, duration of TID, HbA_1c_ after MDI therapy, and HbA1c after CSII therapy.

### Clinic Study Design and Participants

The work was approved by the Medical Ethics Committee of Nanjing Children’s Hospital. We obtained signed informed consent from all participants. All TID cases (n = 240) were recruited from a prospective cohort designed to explore the outcomes of MIT and CSII in Nanjing children’s hospital affiliated to Nanjing Medical University, a tertiary children’s hospital, from 2010 to 2015. Eligibility criteria were as follow. 1) One or more of the following criteria for TID are met: fasting plasma glucose ≥ 7.0mmol/L; 2-h plasma glucose ≥ 11.1 mmol/L following an oral glucose tolerance test (OGTT); diabetes-related symptoms and random plasma glucose ≥11.1mmol/L. 2) All children were 6 years ≤ age < 18 years, without mental disorder. 3) At least one of the three pancreatic islet autoantibodies (gluconic acid decarboxylase antibody, insulin antibody, islet cell autoantibody) is positive, or the results of insulin-C-peptide release test suggest that islet cell function is low (fasting C-peptide < 200 pmol/L). Other types of diabetes, such as type 2 diabetes and special type diabetes, were excluded.

A face-to-face questionnaire interview was conducted at study enrollment to collect demographic information. All participants were randomly divided into two groups: MIT (standard 3 + 1 mode, quick acting insulin injection before three meals combined once long-acting insulin injection) and CSII (insulin pump, basic doses of insulin continuous infusion in 24 h, large doses of insulin infusion before three meals). Of the 240 participants, 52 were excluded due to loss of follow-up. Thus, 188 participants were included in the final analysis, including 112 cases of MIT and 76 cases of CSII.

After the 4-year follow-up, laboratory tests were completed, including HbA_1c_, insulin, C-peptide, kidney function, liver function, blood lipid, 25(OH)D, and thyroid function. TID complications were recorded, which can be divided into acute complications of T1D (diabetic ketoacidosis or diabetic ketosis) and chronic complications (diabetic neuropathy and diabetic eye disease). Growth and pubertal development parameter following MDI and CSII for 4 years were also collected.

### Assessment of Clinical Laboratory Parameter Following MDI and CSII

The fasting blood samples were collected in the morning. Blood urea nitrogen (BUN), creatinine (Cr), aspartate transaminase (AST), alanine transaminase (ALT), high-density lipoprotein (HDL), total cholesterol (TC), and triacylglycerol (TG) were determined by chemiluminescent microparticle immunoassay using the Beckman system (Beckman Coulter, American). Insulin, C-Peptide, total three iodosine adenosine (TT3), free triiodothyronine (FT3), total thyroxine (TT4), free thyroxine (FT4), thyroid-stimulating hormone (TSH), thyroid peroxidase antibody (TPOAb), and thyroglobulin antibody (TGAb) were measured by electrochemiluminescent microparticle immunoassays using the Architect system (Roche GmbH, Germany). HbA_1C_ was measured by high performance liquid chromatography, variant II glycosylated hemoglobin detectors (Bole Company, American). Microalbuminuria (MA), IgG of urinary (IGU), and α 1microglobulin of urinary (A1M) were measured by flow cytometer (Beckman Coulter, American). A 25-hydroxyvitamin D [25(OH)D] was measured by enzymeimmunoassay (Immunodiagnostic Systems Limited, Bolden, UK).

Electromyography (Keypoint 9033A07, Dantec Company, Denmark) and eyes fundus examination (Nidek AFC-300, NIDEK Company, Japan) were performed during hospitalization follow-up.

### Assessment of Growth and Pubertal Development Parameter Following MDI and CSII

After the 4-year follow-up, children’s weight and height were measured, with the accuracy of 0.1 kg and centimeter. Body mass Index (BMI) was calculated using the following formula: BMI = weight (kg)/height (m^2^). BMI categories were based on the World Health Organization classification (19), including <18.5 kg/m^2^, 18.5–24.99 kg/m^2^ and ≥25 kg/m^2^.

We estimated the children’s weight and height at the time of follow-up to Z-score. According to the WHO reference charts, the Z-score is determined by subtracting the median and dividing by the standard deviation using the following formula: Z-score = (XAGE - MAGE)/SDAGE. Age was the age of child, and XAGE is the actual height or weight. MAGE is the median height or weight value at this age, and SDAGE is the standard deviation at this age. Since WHO reference charts present data on height-for-age for children of all ages and weight-for-age for children aged 10 and under, we estimated the height for every child and weight for children aged 10 and under using the Z-score.

The development of breast or genitalia was recorded, and Tanner scale with a five-stage ordinal scale, described by Marshall and Tanner (20, 21), was used to assess the pubertal development of children.

### Statistical Analysis

Meta-analysis was processed in the software Stata 16.0. TID using CSII was set as a reference, and HbA_1c_ levels in MDI was compared to CSII. The heterogeneity of these literature is large (*P* < 0.05, *i*^2^ ≥ 50%), and random effects models will be preferable to fixed effects models (22).

The individual demographic features of 188 TID cases were presented as mean ± SD for continuous variables (i.e., age, birthweight, and clinical laboratory parameter), frequency and percentages (i.e., gender, parity, delivery method, and Tanner stage) for categorical variables. Mean imputation method were imputed to replace missing values of laboratory assessments (23). T-test were used for continuous variables and chi-square test for categorical variables.

We used multiple linear regression models with HbA_1c_, children’s height, weight, and BMI following therapy for 4 years (continuous variables) as dependent variables and different therapy regimens (MDI and CSII) as independent variables. We used logistic regression models to estimate the associations between different therapy regimens (MDI and CSII) and odds of having diabetic ketoacidosis (DKA), abnormal electromyography and abnormal eyes fundus following therapy for 4 years. Model 1 was unadjusted; model 2 was controlled for potential confounders. First, simple linear regression was used to determine whether covariates were selected in our study. According to the standard of p < 0.1, not all covariates included met the requirements. Then, prior knowledge from the scientific literature was used to determine whether covariates were selected for statistical analysis (24). The following variables were included in the final models: children’s age at follow-up, duration of TID, gender, birthweight, parity, and delivery method.

## Results

**Figure 2:**
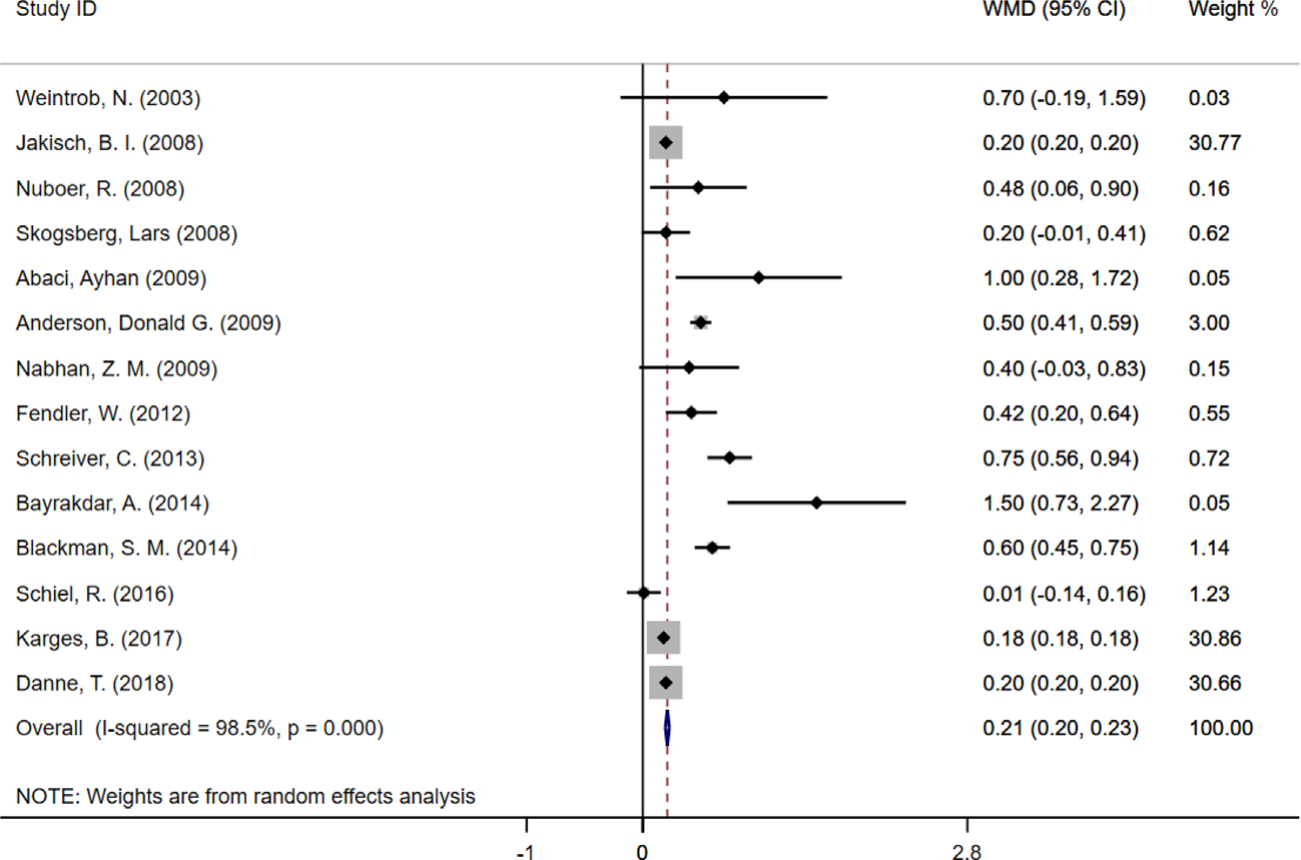
Meta-analysis of HbA1c in type 1 diabetes cases following MDI and CSII therapy.

In meta-analysis, HbA1c were reported in 14 studies involving 69,085 TID cases, including 48,363 MDI, and 20,722 CSII. Characteristics of the literatures were shown in **Table 1**. Compared to CSII in TID cases as a reference, HbA_1c_ levels in MDI (WMD = 0.21, 95% CI: 0.20 to 0.23) were increased significantly (**Figure 2**).

**Table 1:** Characteristics of the literatures included in our meta-analysis.

Author name	Published year	Research time	Region	Sample size (MDI/CSII)	Age(years)	Gender (female/male)	Duration of T1D(years)	MDI HbA1c^1^(%) (means ± SD)	CSII HbA1c(%) (means ± SD)	NOS score
Weintrob, N (9)	2003	lasting 3 months	Israel	12/11	11.8 ± 1.4	13/10	5.8 ± 2.3	8.6 ± 0.8	7.9 ± 1.3	6
Jakisch, B I (11)	2008	January 1995 to June 2006	Germany/Austria	23,649/4,088	10.9	13,314/14,423	3.47	7.7 ± 0.06	7.5 ± 0.05	7
Nuboer, R (6)	2008	lasting 14 months	Holland	19/19	10.0 ± 3.35	21/17	5.15 ± 3.10	7.97 ± 0.78	7.49 ± 0.50	6
Skogsberg, Lars (8)	2008	December 2001 to April 2004	Sweden	38/34	7 to 17	30/42	0.3 ± 0.005	6.7 ± 0.5	6.5 ± 0.4	7
Abaci, Ayhan (5)	2009	2002 to 2006	Turkey	17/17	15.53 ± 1.8	8/9	6.77 ± 4.05	8.71 ± 1.25	7.71 ± 0.84	7
Anderson, Donald G (12)	2009	June 2000 to July 2008	Australia	4,971/959	11.93	266/307	4.3	8.3 ± 1.33	7.8 ± 1.31	8
Nabhan, Z M (7)	2009	November 1999 to April 2003	USA	17/18	3.7 ± 0.8	18/17	1.6 ± 0.6	8.9 ± 0.6	8.5 ± 0.7	6
Fendler, W (13)	2012	January 2002 to December 2010	Poland	223/231	12.13 (8.96–14.64)	199/255	2.44 (1.09–5.37)	7.98 ± 1.38	7.56 ± 0.97	7
Schreiver, C (14)	2013	January 2010 to December 2010	Germany	26/22	12.9 ± 3.3	26/22	5.3 ± 3.7	9.03 ± 0.42	8.28 ± 0.25	6
Bayrakdar, A (15)	2014	2003	Lebanon	18/18	19.2 ± 2.6	16/20	9.1 ± 5.1	8.9 ± 1.4	7.4 ± 0.9	6
Blackman, S M (16)	2014	September 2010 to August 1, 2012	USA	337/332	1 to 6	285/384	1 to 5	8.5 ± 1.1	7.9 ± 0.9	7
Schiel, R (17)	2016	April 1 2004 to October 31 2010	Germany	707/194	11.5 ± 4.0	468/433	4.0 ± 3.6	7.45 ± 1.21	7.44 ± 0.82	7
Karges, B (3)	2017	January 2011 to December 2015	Germany/Austria/Luxembourg	16,460/14,119	14.1 ± 4.0	14,372/16,207	7.0 ± 3.9	8.22 (8.18–8.25)^*^	8.04 (8.00–8.07)^*^	8
Danne, T (18)	2018	January 1, 2013 to January 1, 2017	German/Austrian	1,869/660	11.87 ± 3.4	1,140/1,389	3.87 ± 3.33	7.7 ± 0.02	7.5 ± 0.03	8

MDI, Multiple daily injections; CSII, Continuous subcutaneous insulin infusion.

^*^Mean and 95%CI.

**Figure 3:**
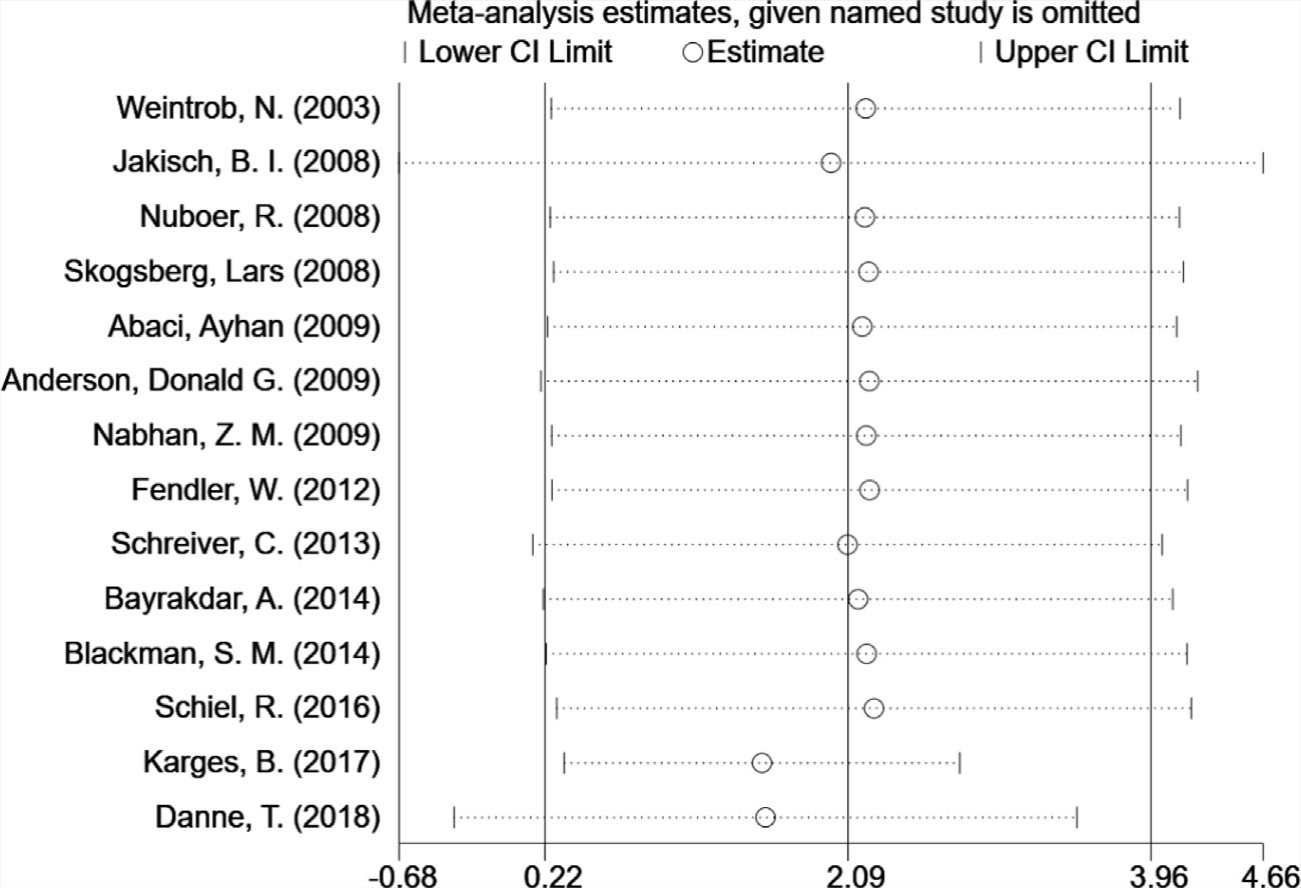
Sensitivity analysis of HbA1c in type 1 diabetes cases following MDI and CSII therapy.

We used a one-by-one elimination method for sensitivity analysis and chose a random effects model. The results showed that the pooled ES values before and after the exclusion of a study were essentially the same as the 95% confidence intervals, indicating that the original meta-analysis were reliable (**Figure 3**). In addition, we did not observe significant publication bias, using Begg’s test and funnel plots (Z = 0.88, P = 0.381).

In 188 clinical cases, overall individual demographic features were described in **Table 2**. Mean age at recruitment was 7.55 (SD 2.91) years. Mean age at follow-up was 11.75 (SD 2.53) years. Duration of TID was 4.23 (SD 2.61) years. 50.53% (n = 95) of them were boys. In addition, 112 cases used MIT and 76 used CSII. No significant differences were observed between MIT and CSII in children’s age at recruitment, children’s age at follow-up, duration of TID, gender, birthweight, parity, delivery method, and feeding method.

**Table 2:** The individual demographic features of 188 type 1 diabetes cases.

Characteristic	Total (N = 188)	MIT (N = 112)	CSII (N = 76)	*p*
Age at recruitment (years)	7.55 ± 2.91	7.39 ± 2.65	7.76 ± 3.22	0.410
Age at follow-up (years)	11.75 ± 2.53	11.58 ± 2.50	12.00 ± 2.56	0.259
Duration (years)	4.23 ± 2.61	4.24 ± 2.38	4.21 ± 2.93	0.951
Gender				
boy	95 (50.53)	58 (51.79)	37 (48.68)	0.676
girl	93 (49.47)	54 (48.21)	39 (51.32)	
Birthweight (kg)	3.45 ± 0.47	3.45 ± 0.44	3.45 ± 0.50	0.944
Parity				
1	122 (64.89)	65 (58.04)	57 (75.00)	0.082
2	50 (26.60)	34 (30.36)	16 (21.05)	
3	9 (4.79)	7 (6.25)	2 (2.63)	
4	7 (3.72)	6 (5.36)	1 (1.32)	
Delivery method				
Vaginal birth	110 (58.51)	62 (55.36)	48 (63.16)	0.287
Caesarean section	78 (41.49)	50 (44.64)	28 (36.84)	
Feeding methods within 6 months				
Artificial feeding	31 (16.49)	15 (13.39)	16 (21.05)	0.165
Non-artificial feeding	157 (83.51)	97 (86.61)	60 (78.95)	

**Table 3** showed the laboratory assessments following MDI or CSII for about 4 years. The children treated with CSII had lower concentration of HbA1c, ALT, AST, HDL, TPOAb, and A1M than those of treated with MDI (*P* < 0.05). There were no significant differences between the effects of MIT and CSII on acute and chronic complications, including diabetic ketoacidosis, diabetic ketosis, abnormal electromyography, and abnormal eyes fundus.

**Table 3:** Clinical laboratory parameter following MDI or CSII therapy for about 4 years.

Characteristic	Total (N = 188)	MIT (N = 112)	CSII (N = 76)	*p*
HbA1c (%)	8.40 ± 2.21	8.71 ± 1.80	7.93 ± 2.08	**0.012**
Insulin (mU/L)	4.03 ± 34.89	4.53 ± 5.56	3.30 ± 3.61	0.091
C-Peptide (nmol/L)	0.05 ± 0.08	0.05 ± 0.07	0.06 ± 0.09	0.498
CR (umol/L)	45.06 ± 11.65	45.03 ± 12.03	45.10 ± 11.15	0.969
BUN (mmol/L)	4.92 ± 1.17	4.94 ± 1.25	4.89 ± 1.06	0.774
ALT (U/L)	13.44 ± 7.09	14.56 ± 7.64	11.79 ± 5.86	**0.008**
AST (U/L)	19.57 ± 8.28	20.71 ± 9.83	17.88 ± 4.81	**0.021**
HDL (mmol/L)	1.53 ± 0.33	1.57 ± 0.34	1.47 ± 0.29	0.056
TC (mmol/L)	4.14 ± 0.98	4.13 ± 0.98	4.15 ± 1.00	0.856
TG (mmol/L)	0.98 ± 0.63	1.03 ± 0.72	0.90 ± 0.44	0.146
25(OH)D (nmol/L)	45.34 ± 14.00	45.36 ± 15.56	45.31 ± 11.41	0.980
TT3 (nmol/L)	2.00 ± 0.42	2.02 ± 0.42	1.97 ± 0.43	0.454
FT3 (pmol/L)	5.87 ± 1.03	5.95 ± 1.07	5.76 ± 0.95	0.204
TT4 (nmol/L)	95.70 ± 20.11	95.59 ± 22.15	95.87 ± 16.79	0.925
FT4 (pmol/L)	17.83 ± 10.37	17.22 ± 2.61	18.74 ± 16.02	0.326
TSH (uIU/ml)	3.62 ± 1.74	3.54 ± 1.94	3.75 ± 1.40	0.421
TPOAb (IU/ml)	63.95 ± 142.39	81.66 ± 162.30	37.86 ± 102.03	**0.038**
TGAb (IU/ml)	153.0± 515.54	149.5 ± 464.64	158.0 ± 585.78	0.912
MA (mg/L)	8.04 ± 12.02	8.44 ± 13.76	7.46 ± 8.93	0.586
IGU (mg/L)	5.26 ± 3.57	5.17 ± 3.20	5.38 ± 4.08	0.705
A1M (mg/L)	6.33 ± 4.43	6.88 ± 5.52	5.52 ± 1.65	**0.039**
DK/DKA				
No	180 (95.74)	108 (96.43)	72 (94.74)	0.647
DK	3 (1.60)	1 (0.89)	2 (2.63)	
DKA	5 (2.66)	3 (2.68)	2 (2.63)	
Electromyography				
Normal	110 (58.51)	64 (57.14)	46 (60.53)	0.644
Abnormal	78 (41.49)	48 (42.86)	30 (39.47)	
Eyes fundus				
Normal	186 (98.94)	110 (98.21)	76 (100.00)	0.242
Abnormal	2 (1.06)	2 (1.79)	0 (0.00)	

HbA1c, Glycosylated hemoglobin; Cr, Creatinine; BUN, Blood urea nitrogen; ALT, Alanine aminotransferase; AST, Aspartate aminotransferase; HDL, High-density lipoprotein; TC, Total cholesterol; TG, Triacylglycerol; 25(OH)D, 25-hydroxyvitamin D; TT3, Total three iodosine adenosine; FT3, Free three iodine armor gland original acid; TT4, Total thyroxine; FT4, Free thyroxine; TSH, Thyroid-stimulating hormone; TPOAb, Thyroid peroxidase antibody; TGAb, Thyroglobulin antibody; MA, Microalbuminuria; IGU, IgG of urinary; A1M, α 1microglobulin of urinary; DKA, Diabetic ketoacidosis; DK, Diabetic ketosis.

Statistically significant results (p < 0.05) are bolded.

**Table 4** showed the growth and pubertal development parameter following MDI and CSII therapy for about 4 years. Children treated with CSII for about 4 years showed increased weight levels and weight Z-score (P < 0.05), as well as trends of increased height levels and height Z-score in TID using MDI (P > 0.05), while there was no difference in the status of BMI and pubertal development following the two therapy regimens.

**Table 4:** Growth and pubertal development parameter following MDI and CSII therapy for about 4 years.

Characteristic	Total (N = 188)	MIT (N = 112)	CSII (N = 76)	*p*
Height (cm)	150.54 ± 13.34	149.13 ± 14.78	152.62 ± 10.63	0.078
Height Z-score^a^	0.71 ± 1.12	0.70 ± 1.12	0.79 ± 1.13	0.523
Weight (kg)	41.35 ± 11.89	39.73 ± 11.84	43.75 ± 11.63	**0.022**
Weight Z-score^a^	0.63 ± 0.81	0.43 ± 0.70	0.94 ± 0.87	**0.010**
BMI (kg/m^2^)				
<18.5	127 (67.55)	80 (71.43)	47 (61.84)	0.325
18.5–24.99	56 (29.79)	30 (26.79)	26 (34.21)	
>25	5 (2.66)	2 (1.79)	3 (3.95)	
Tanner stage				
Phase I	54 (28.72)	38 (33.93)	16 (21.05)	0.116
Phase II	57 (30.32)	30 (26.79)	27 (35.53)	
Phase III	55 (29.26)	35 (31.25)	20 (26.32)	
Phase IV	19 (10.11)	8 (7.14)	11 (14.47)	
Phase V	3 (1.60)	1 (0.89)	2 (2.63)	

a Since WHO reference charts present data on height-for-age for children of all ages and weight-for-age for children aged 10 and under, we estimated the height for every child and weight for children aged 10 and under using the Z-score. Statistically significant results (p < 0.05) are bolded.

**Tables 5** and **6** showed the associations between different therapy regimens with key outcomes. Specifically, children’s HbA_1c_ was 0.67 (95% CI −1.28, −0.05) % lower in children following CSII for about 4 years compared to children following MDI in multivariable regression models with adjustment for potential confounders (children’s age at follow-up, duration of TID, gender, birthweight, parity, and delivery method). Children using CSII therapy weighed 2.31 kg heavier (95% CI 0.59, 4.04) than children in MDI group in the adjusted model. CSII did not increase the odds of long-term complications, such as diabetic ketoacidosis, diabetic ketosis, and abnormal electromyography.

**Table 5:** Associations between different therapy methods with children’s HbA1c, height, weight, and BMI.

Therapy method	Model 1^a^β (95%CI)	*p*	Model 2^b^β (95%CI)	*p*
Children’s HbA1c (%) following therapy				
MDI	ref	ref		
CSII	−0.78 (−1.38, −0.17)	**0.012**	−0.67 (−1.28, −0.05)	**0.033**
Children’s Height (cm) following therapy				
MDI	ref	ref		
CSII	3.49 (−0.40, 7.38)	0.078	1.79 (−0.80, 4.38)	0.173
Children’s Weight (kg) following therapy				
MDI	ref	ref		
CSII	4.03 (0.58, 7.47)	**0.022**	2.31 (0.59, 4.04)	**0.009**

Statistically significant results (p < 0.05) are bolded.

MDI, Multiple daily injections; CSII, Continuous subcutaneous insulin infusion. Model 1 was unadjusted model; Model 2 was adjusted for children’s age at follow-up, duration of type 1 diabetes, gender, birthweight, parity, and delivery method.

**Table 6:** Associations between different therapy methods with complications.

Therapy method	Model 1^a^ OR (95%CI)			*p*	Model 2^b^ OR (95%CI)		*p*
DK or DKA following therapy							
MDI		ref				ref	
CSII		1.5 (0.36, 6.19)	0.575			2.33 (0.52, 10.45)	0.271
Electromyography							
MDI		ref				ref	
CSII		0.87 (0.48, 1.57)	0.644			0.82 (0.43, 1.55)	0.536
Eyes fundus							
MDI		ref				ref	
CSII		–	–			–	–

MDI, Multiple daily injections; CSII, Continuous subcutaneous insulin infusion; DKA, Diabetic ketoacidosis; DK, Diabetic ketosis. Model 1 was unadjusted model; Model 2 was adjusted for children’s age at follow-up, duration of type 1 diabetes, gender, birthweight, parity, and delivery method.

## Discussion

Glycemic control of TID in children with different therapy regimens has always been the focus in pediatric endocrinology. Previous observational study has proposed that CSII can effectively control glucose (3, 11, 18), but causal relationships between different therapy regimens and long-term safety outcomes remains controversial (25, 26). We completed a design combining meta-analysis and prospective cohort study, and confirmed CSII could decrease the HbA_1c_ better than MIT in pediatric T1D. In our cohort data, no high rates of long-term complications were observed in CSII, and at the same time, there was a better effect of CSII for children growth development.

HbA_1c_ is a key indicator for evaluating glycemic control. According to the guideline published by National Institute for Health and Care Excellence, it was recommended that children aged ≥ 12 years who cannot achieve HbA_1c_ < 8.5% should be offered CSII therapy. Children aged < 12 years should be offered CSII therapy from diagnosis of T1D if MDI is considered inappropriate (27). Fourteen literature were included in our meta-analysis with 69,085 TID cases, of which most are observational data from clinical registry system or randomized clinical trials, and the data suggested that CSII may be better than MIT for glycemic control. The results of our cohort study confirmed the conclusion of the meta-analysis. Hence, in terms of glycemic control, our results reveal that CSII took an advantage over MIT.

Therapeutic safety was our secondary focus on the outcomes of CSII in our cohort. Previous research suggested that CSII had the risk of elevated ketoacidosis in children below 12 years, while might reduce ketoacidosis risk in adolescents (28). However, Karges B. indicated that CSII was associated with a lower rate of ketoacidosis (3), and he pointed out that different rates in the use of CSII in different studies were responsible for the discrepancy. In our cohort, the utilization rate of CSII was 40.4%, and after 4 years therapy, there was no difference in the incidence of ketoacidosis or ketosis between CSII and MIT. We also followed up nephropathy consequences and found CSII leading to lower A1M levels, a kind of urinary microalbuminuria, maybe indicating a protective effect on renal complications. In addition, no difference was found in peripheral nerve and fundus consequences between CSII and MIT.

Further, growth and pubertal development were our third focus on the outcomes of CSII in our cohort. One observation study investigated that compared to control children, children with T1D were taller and heavier at the time of the initial diagnosis throughout childhood (29), while another longitudinal study showed retardation in physical growth and delayed age at menarche in girls and full pubertal maturation in boys were in T1D children (30). In our study, the children treated with CSII for about 4 years showed significant increased weight and weight Z-score and trends of increased height levels and height Z-score, perhaps indicating a better effect of CSII for children growth development. On the other hand, there was no difference in the status of obesity and thin as well as pubertal development following the two therapy regimens.

A major advantage of this present study was that we combined meta-analysis and prospective cohort study. That is to exhibit the background part of our paper in the way of data presentation, as well as obtain the clues from the systematic evaluation and analysis of previous literature. We further verified causal relationship between insulin therapy regimens and outcomes in our prospective cohort, which avoided the limitation of single center and small sample size, and made the analysis result more comprehensive and reliable. Our cohort study focused not only on glycemic control of different therapy regimens, but also on long-term complications and growth and pubertal development, understudied outcomes, with T1D duration longer than 4 year throughout childhood. Another potential advantage was that the individual age and duration of different therapy regimens matched well, and was considered in the analyses. Thus, our findings could be important, especially for care on growth and development in children with T1D.

Nevertheless, this study had some limitations. First, the sample size of the single center cohort study is small, and our results need to be validated by a larger sample size study. In addition, studies report that factors such as family socioeconomic status (SES) and children’s nutrition are correlated with glycemic control (31–33), however, we lack some important covariables in the regression model due to the insufficient response rate of participants, so the overall stability of the model is not strong enough. HbA1c is the gold standard indirect measure of glucose control and it estimates the glycemic exposure over the last three months prior to sampling. However, glucose levels can undergo large fluctuations secondary to other factors and this glucose metric, used alone, may be insufficient to define a good control. Other parameters that we lack, such as severe hypoglycemic episodes, time in range (TIR), time below range (TBR), and time above range (TAR), could also be useful. Furthermore, during the period from T1D diagnosis to 4-year follow-up, we conducted multi-time follow-up, however, due to the high rate of lost follow-up at some time points, only the single time point of 4 years was retained as the final analysis. These individuals will be followed up for further study in the future.

## Conclusions

Altogether, in this present study of children with T1D, compared to MIT, CSII might be associated with better glycemic control and better effects for children growth development, and no higher risks of long-term complications and delayed pubertal development were observed in CSII, nevertheless, our conclusions need to be validated by a larger sample size study.

## Data Availability Statement

The original contributions presented in the study are included in the article/supplementary material. Further inquiries can be directed to the corresponding author.

## Ethics Statement

The studies involving human participants were reviewed and approved by IRB of Children’s Hospital of Nanjing Medical University. Written informed consent to participate in this study was provided by the participants’ legal guardian/next of kin.

## Author Contributions

The authors such as XW, XZ, and DC contributed equally to this work. They collected, analyzed data, and wrote articles. All authors contributed to the article and approved the submitted version.

## Funding

This work was supported by the National Natural Science Foundation of China (81803259).

## Conflict of Interest

The authors declare that the research was conducted in the absence of any commercial or financial relationships that could be construed as a potential conflict of interest.
